# Perilesional Inflammation in Neurocysticercosis - Relationship Between Contrast-Enhanced Magnetic Resonance Imaging, Evans Blue Staining and Histopathology in the Pig Model

**DOI:** 10.1371/journal.pntd.0004869

**Published:** 2016-07-26

**Authors:** Carla Cangalaya, Javier A. Bustos, Juan Calcina, Ana Vargas-Calla, Diego Suarez, Armando E. Gonzalez, Juan Chacaltana, Cristina Guerra-Giraldez, Siddhartha Mahanty, Theodore E. Nash, Hector H. García, Cysticercosis Working Group in Peru

**Affiliations:** 1 Laboratorio de Inmunopatología en Neurocisticercosis, Facultad de Ciencias y Filosofía, Universidad Peruana Cayetano Heredia, Lima, Peru; 2 Facultad de Medicina Humana, Universidad Nacional Mayor de San Marcos, Lima, Peru; 3 Unidad de Cisticercosis, Instituto Nacional de Ciencias Neurológicas, Lima, Peru; 4 Facultad de Medicina Veterinaria, Universidad Nacional Mayor de San Marcos, Lima, Peru; 5 Facultad de Medicina, Universidad Peruana Cayetano Heredia, Lima, Peru; 6 Departamento de Diagnóstico por imágenes, Instituto Nacional de Ciencias Neurológicas, Lima, Peru; 7 Departamento de Ciencias Celulares y Moleculares, Facultad de Ciencias y Filosofía, Universidad Peruana Cayetano Heredia, Lima, Peru; 8 Laboratory of Parasitic Diseases, National Institute of Allergy and Infectious Diseases, National Institutes of Health, Bethesda, Maryland, United States of America; Albert Einstein College of Medicine, UNITED STATES

## Abstract

**Background:**

Disease manifestations in neurocysticercosis (NCC) are frequently due to inflammation of degenerating *Taenia solium* brain cysts. Exacerbated inflammation post anthelmintic treatment is associated with leakage of the blood brain barrier (BBB) using Evans blue (EB) staining. How well EB extravasation into the brain correlates with magnetic resonance imaging (MRI) using gadolinium (Gd) enhancement as a contrast agent and pericystic inflammation was analyzed in pigs harboring brain cysts of *Taenia solium*.

**Methodology/Principal Findings:**

Three groups of 4 naturally infected pigs were assessed. The first and second groups were treated with both praziquantel plus albendazole and sacrificed two and five days post treatment, respectively. A third untreated group remained untreated. Pigs were injected with EB two hours prior to evaluation by Gd-enhanced T1-MRI, and euthanized. The EB staining for each cyst capsule was scored (EB grades were 0: 0%; 1: up to 50%; 2: over 50% but less than 100%; 3: 100%). Similarly, the Gd enhancement around each cyst was qualitatively and quantitatively scored from the MRI. The extent of pericystic inflammation on histology was scored in increasing severity as IS1, IS2, IS3 and IS4. Grade 3 EB staining and enhancement was only seen in treated capsules. Also, treated groups had higher Gd intensity than the untreated group. Grades of enhancement correlated significantly with Gd enhancement intensity. EB staining was correlated with Gd enhancement intensity and with IS4 in the treated groups. These correlations were stronger in internally located cysts compared to superficial cysts in treated groups.

**Significance:**

EB staining and Gd enhancement strongly correlate. The intensity of enhancement determined by MRI is a good indication of the degree of inflammation. Similarly, EB staining highly correlates with the degree of inflammation and may be applied to study inflammation in the pig model of NCC.

## Author Summary

Neurocysticercosis (NCC) is a frequent parasitic infection of the human brain in developing countries. The symptomatology of human NCC after antiparasitic treatment is generally related to inflammation. The presence and degree of enhancement after intravascular injection of the contrast agent gadolinium in magnetic resonance imaging (MRI) is commonly considered an evidence of blood brain barrier (BBB) leakage. Experimentally, the presence and degree of extravasation of Evans blue (EB) after intravascular injection into the tissues of the brain is a direct measure of blood brain barrier leakage. The BBB leakage of gadolinium in neurocysticercosis is commonly used as an indirect measure of inflammation but has never been experimentally proven. Here we evaluated the relationship between contrast T1-MRI, EB staining and histology findings in naturally infected pigs. There was a strong correlation between EB staining, contrast MRI and histopathology findings after antiparasitic treatment. This correlation was stronger when cysts were internally located in the brain than in superficial cysts partly located in the subarachnoid space (meninges). Contrast-enhanced MRI is a non invasive tool used in diagnosis and follow up of NCC patients. This study shows that the use of EB staining allows for the same conclusions as when using MRI post-treatment, and that both techniques correlate with histopathology findings. These results support the use of EB staining to study NCC using the porcine model as well as validate MRI enhancement to assess brain inflammation in patients.

## Introduction

Neurocysticercosis (NCC), infection of the brain by the larval stage of the parasite *Taenia solium*, is a common cause of epilepsy in endemic countries [1]. Imaging studies are essential to diagnose NCC. Magnetic resonance imaging (MRI) is more sensitive than computed axial tomography (CT), providing better anatomical differentiation, superior visualization of small lesions, edema, vascular enhancement and tissue changes; CT scan is more sensitive for the detection of calcified lesions [2, 3].

Cysts that have formed in the brain parenchyma can remain quiescent for a period of months to years. At some point, either as a result of the natural course of disease or because of cysticidal treatment, the host mounts a focal inflammatory immune response to the cyst resulting in parasite degeneration [1]. A contrast-enhanced MRI at this time shows a hyperdense ring in the adjacent capsule surrounding the cysticercus, reflecting blood brain barrier (BBB) disruption [4]. Previous experiments by our group using a *T*. *solium* naturally-infected pig model [5, 6] measured the degree of capsular inflammation and the extravasation of Evans blue dye (EB) into the inflamed capsules of degenerating cysts. The presence of EB capsular staining, a measure of BBB disruption, correlates with the degree of pericystic inflammation [6].

Among imaging techniques, fluid-attenuated inversion recovery (FLAIR) and contrast-enhanced T1-MRI using gadolinium as contrast agent are the most useful for diagnostics and follow up, as they provide more detail about the stage of the inflammatory response and the evolution of the damage suffered by the parasite [3]. In the evaluation of human NCC, the degree of enhancement in the contrast T1-MRI protocol is commonly assumed to be a measure of the amount of inflammation present in specific lesions [4]. Since both contrast enhancement and EB staining reflect BBB dysfunction, we assessed whether these markers correlate between themselves and to pericystic brain inflammation as determined on histology. A significant correlation would support the use of enhancement on MRI as a measure of inflammation in swine and by analogy to human NCC.

## Materials and Methods

### Study design and animals

We compared pericystic EB staining, gadolinium enhancement on MRI, and histological findings following cysticidal treatment of pigs naturally infected with *T*. *solium* brain cysts. An individual cyst was considered the unit of analysis for this study. Twelve pigs naturally infected with *T*. *solium* cysts from endemic Peruvian highland villages, confirmed by positive tongue examination [7], were imaged by MRI to confirm brain infection and then randomly divided in three groups of four pigs each. One group remained untreated as a untreated, and the other two groups were treated with albendazole (Zentel, GlaxoSmithKline, Peru) at 15 mg/kg daily until sacrifice plus a single day treatment of praziquantel (Helmiben, Farmindustria, Peru) at 75 mg/kg divided into three doses of 25 mg/kg every two hours on the first day [7]. Pigs in the treatment groups were sacrificed two (n = 4) and five (n = 4) days after treatment.

### Post-treatment procedures

Immediately before sacrifice, all pigs were anesthetized with an intramuscular injection of a mixture of ketamine (Ket-A-100 50 mg/kg, Agrovet Market SA, Peru) and xylazine (Dormi-Xyl 2mg/kg, Agrovet Market SA, Peru) through a catheter inserted into the marginal ear artery and kept latent by very slow normal saline drip. Then they were infused through the ear catheter with EB dye first [5], and after 45 minutes with Gadolinium diethylene triaminopentaacetic acid (Gd-DTPA) at 0.1 mmol/kg for contrast-enhanced brain MRI. Shortly after the MRI, the pigs were perfused intraaortally for 15 minutes with normal saline solution using a peristaltic pump and euthanized with a lethal IM dose of 120 mg/kg of pentobarbital. The pigs’ brains were retrieved at necropsy and examined macroscopically and by histology.

### Evans Blue staining

A 2% EB (Sigma–Aldrich, St. Louis, MO) solution in normal saline was administered by the EB catheter as reported [5, 6] and allowed to circulate for 2 hours under additional sodium pentobarbital sedation (Halatal, AgrovetMarket SA Peru, at 25 mg/kg every 45 min).

#### Grading of EB staining

**Fig 1:**
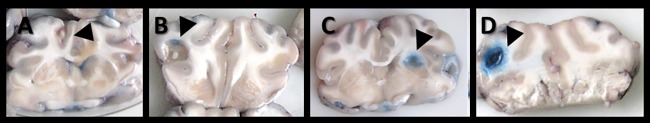
Typical EB stages. Arrows show the capsule and cyst for each grade. A) Grade 0, B) Grade 1, C) Grade 2 and D) Grade 3.

Increase of BBB permeability was assessed by the degree of extravasation of EB into the host tissue around the cysticercus (capsule). Cyst capsules were scored qualitatively according to distribution of the color per cyst. EB staining grades were 0: 0%; 1: up to 50% of the capsule was blue; 2: over 50% of the capsule was blue; 3: 100% of the capsule was blue (Fig 1).

### MRI examinations

Sedated pigs were placed on a surgical table in a side-lying position. Pre and post-contrast MRI were performed on a 3-Tesla scanner (Philips Achieva, Best, The Netherlands) including axial, coronal, and sagittal TFE (Turbo field echo). T1-weighted gradient-echo images were taken with 1–4 mm section thickness, 7 milliseconds (ms) of repetition time, 4 ms echo time, 8° flip angle, 270kHz pixel bandwidth and matrix = 256–480 pixels.

#### MRI Imaging interpretation and analysis

**Fig 2:**
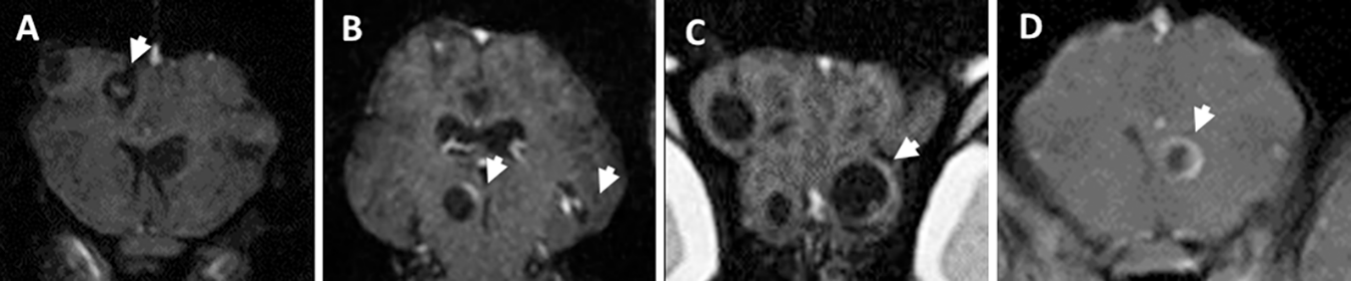
Typical gadolinium (Gd) enhancement grades. Arrows show the capsule and cyst for each grade. A) Grade 0, B) Grade 1, C) Grade 2 and D) Grade 3.

Post-contrast MR images were used to examine and classify cyst lesions using qualitative and quantitative measures. Positive enhancement is defined as a post-contrast hyperintense signal along the edge of a cyst. Qualitative evaluation was based upon the percent of cyst involved in the signal; all consecutive images (1–10) that belonged to each cyst were individually graded in a way similar as used for EB staining: 0 (no enhancement); 1 (up to 50% of the capsule enhanced); 2 (over 50% of the capsule enhanced), or 3 (100% of the capsule enhanced) (Fig 2). A global, single grade was assigned to each cyst by the following criteria: grades 0 and 3 required all of its images to be grade 0 or 3; grade 1 was given when at least one image was grade 1 but none was grade 2 or 3: cysts with images in any other combination were grade 2.

A quantitative measurement of Gd enhancement intensity was also built using the open access image-processing program FIJI (FIJI/ImageJ, NIH, Bethesda, Maryland, USA) to convert digital imaging and communication in medicine (DICOM) scan images to tagged image file format (TIFF). Brightness and contrast in the TIFF scan images were first adjusted to define and delineate pericystic lesion areas, and then the gray values in each pixel within these selections were normalized using the percentage of a continuous scale from 0 (black) to 256 (brightest). Then, the average gray value from all pixels within the selection of each image was calculated. Finally, we calculated the average of intensity of Gd enhancement in all pericystic section images in order to obtain a single value for each cyst using R program v3.2.2 [8].

### Brain examinations

Immediately upon extraction, brains were placed on dry ice, which helped with the slicing of the samples into 1-cm coronal sections. The anterior and posterior surfaces of each section were photographed.

#### Cyst location

Cysticerci were characterized as superficial or deep cysts. Superficial cysts are those in a cortical location with a region exposed to meninges and another embedded in parenchyma, mainly in gray matter [9]. Deep cysts were those totally embedded into the brain parenchyma (white and gray matter), with close proximity to the brain center (coordinates x, y, z = 0, 0, 0).

#### Histology

All capsules were excised from the brains. And each biopsy from the right brain hemispheres was separately fixed in 10% neutral buffered formalin (3.7% formaldehyde in PBS, pH 7.2). Fixed biopsies were embedded in paraffin and sliced in coronal 4 μm-thick sections. Conventional hematoxylin-eosin stains were performed on every slide and two sections of the same cyst were examined with conventional light microscopy. Microphotographs taken with 15X magnification were taken with a Carl Zeiss stereoscope and assembled using AxioVision software (Rel. 4.6, Zeiss, Oberkochen, Germany) to obtain a single large image (“cyst map”). Biopsies from the left hemispheres were saved for parallel studies.

#### Inflammatory response

**Fig 3:**
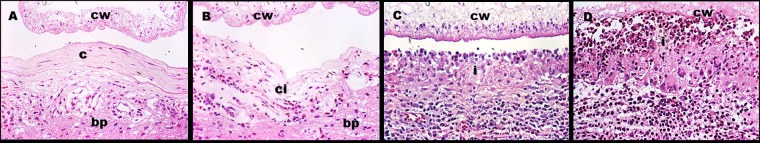
Inflammatory stages. cw = Cyst wall, c = collagen, i = immune cells infiltrate, ci = collagen plus immune cells infiltrate, bp = brain parenchyma. **A** shows IS1 with few immune cells into the collagen layer. **B** shows IS2 where the immune cells are more abundant. **C** shows IS3 where the number of immune cells were increased and ordered in layers. **D** shows IS4 where multinucleated giant cells are increased, the cyst wall seen damaged and a near layer of eosinophils.

As in previous studies, the areas of inflammatory response around the cyst were categorized in inflammatory stages (IS) 1 to 4, where IS1 was a thin layer of collagen with scarce or null immune cells, IS2 was a thicker layer of collagen with more non organized immune cells, IS3 was a typical granulomatous reaction with abundant immune cells distributed in layers (an epithelioid cells or eosinophil-rich layer near to the cyst wall with few multinucleated giant cells) and IS4 was similar to IS3 but with higher numbers of eosinophils (identified by their red staining granules) and with an increase of multinucleated giant cells distributed in a layer close to the cyst wall with the parasite structure clearly damaged (Fig 3). Since the IS around a cyst was usually not homogeneous, the proportions (percents) of the perimeter presenting each inflammatory stage were recorded using the cyst map [6].

### Statistical analysis

Spearman´s rank correlation was used to evaluate the correlation between the qualitative and quantitative measure of Gd enhancement and the relationship between the grades of EB staining (ordinal variable) with quantitative measure of Gd enhancement in each treatment group, and the correlation between each inflammatory stage (IS1 to IS4 extension expressed in percentage as a four continuous variables) with the grades of EB staining and quantitative enhancement measure. The non-parametric Wilcoxon-Mann Whitney test was used to compare Gd enhancement intensity and EB grades between the different locations of the cyst in each individual treatment group. All the analyses were performed using the R v3.2.2 and graphs were created using ggplot package [8]. Differences were considered significant at p<0.05.

### Ethical statement

The study was conducted in accordance with the National Institutes of Health/AALC guidelines, and was reviewed and approved by the Institutional Animal Care and Use Committee Animal Ethics of the Universidad Peruana Cayetano Heredia (assurance Number: A5146-0).

## Results

### Cyst distribution per animal and per brain hemisphere

A total of 328 brain cysts from 12 naturally infected pigs were assessed in this study. The parasitic load per pig brain was widely distributed, ranging from 1 to 45 cysts in the untreated group, from 10 to 29 cysts in PZQ+ABZ 2d and from 4 to 152 cysts in the group allocated to PZQ+ABZ 5d. Pigs in the PZQ+ABZ 5d group had more cysts (n = 192) than did pigs in the untreated group (n = 73) or in the PZQ+ABZ 2d group (n = 63). The distribution of cysts in both hemispheres was similar in each pig, a total of 165 cysts were found in the right hemispheres of study pigs and 163 cysts were found in the left hemispheres. All brain cysts (n = 328) were used for macroscopic assessment of Evans blue uptake and also for Gd enhancement on MRI (Table 1).

**Table 1:** Cyst numbers and distribution in study pigs.

Characteristics	Untreated (n = 4)	PZQ+ABZ 2d (n = 4)	PZQ+ABZ 5d (n = 4)	Total (n = 12)
**Brain cyst load (per pig)**	73 (1, 11, 16, 45)	63 (10, 11, 13, 29)	192 (4,13, 23, 152)	328
**Total cyst distribution (right / left hemisphere distribution per pig)**	30/43 (0/1, 6/5, 5/11, 19/26)	34/29 (7/3, 5/6, 7/6,15/14)	101/91 (3/1, 9/4, 13/10, 76/76)	165/163

### Evans Blue staining

Cysts with clear capsules (grade 0) were seen only in untreated animals; whereas EB grade 3 capsules (completely blue) were seen only in animals treated with antiparasitic drugs (Table 2). Capsules from treated groups had significantly more EB staining than untreated capsules (PZQ+ABZ 2d versus untreated group, p = 0.02 and PZQ+ABZ 5d versus untreated group, p< 0.001). However the capsules from PZQ+ABZ 2d had more EB staining than those in the PZQ+ABZ 5d group (p< 0.001).

**Table 2:** Grades of Evans blue staining in brain cysts by treatment group. Values represent numbers of brain cyst capsules in each stratum.

		Untreated	PZQ+ABZ 2d	PZQ+ABZ 5d
**Evans blue staining (Cyst capsules, %)**	*Grade 0*	6 (8%)	0 (0%)	0 (0%)
	*Grade 1*	34 (47%)	5 (8%)	10 (5%)
	*Grade 2*	33 (45%)	19 (30%)	144 (75%)
	*Grade 3*	0 (0%)	39 (62%)	38 (20%)

p = 0.02, p< 0.001 and p< 0.001 for comparisons of EB staining between: PZQ+ABZ 2d versus untreated group, PZQ+ABZ 5d versus untreated group, and PZQ+ABZ 5d vs PZQ+ABZ 2d, respectively.

The apparent decrease in EB staining from day 2 to day 5 (proportional increase in EB grade 2 capsules with fewer grade 3 capsules) may be due to one pig contributing 128 out of the 144 grade 2 capsules in day 5 (S1 Table).

### Gadolinium enhancement in MRI

Similarly to the EB staining findings, cyst capsules without Gd enhancement (grade 0 of enhancement) were seen only in the untreated group, whereas completely enhanced capsules (grade 3) were seen only in treated animals. Capsules in treated groups had more Gd enhancement than capsules from the untreated group (p< 0.001), however there were marginal differences between both treated groups (p = 0.06) (Table 3). Again, although the data suggest a maximum effect on day 2, this may have been caused by many grade 2 cysts in the PZQ+ABZ 5d group that came from the same pig (S2 Table). Quantitative measurements of Gd enhancement by cyst were highly correlated to the above-described qualitative assessment (Table 4). From here on, all analyses shown refer to the quantitative measurement of enhancement, which is less subjective.

**Table 3:** Qualitative grade assessment of Gd enhancement in MRI of brain cysts by treatment group. Values represent numbers of brain cyst capsules in each stratum.

		Untreated	PZQ+ABZ 2d	PZQ+ABZ 5d
**Gadolinium enhancement (Cyst capsules, %)**	*Grade 0*	4 (6%)	0 (0%)	0 (0%)
	*Grade 1*	58 (79%)	10 (16%)	22 (11%)
	*Grade 2*	11 (15%)	39 (62%)	161 (84%)
	*Grade 3*	0 (0%)	14 (22%)	9 (5%)

p< 0.001, p< 0.001 and p = 0.06 for comparisons of Gd enhancement between PZQ+ABZ 2d versus untreated group, PZQ+ABZ 5d versus untreated group, and PZQ+ABZ 5d vs PZQ+ABZ 2d, respectively.

**Table 4:** Correlation between qualitative and quantitative Gd enhancement measures in brain cysts by treatment group. Values represent medians and ranges of quantitative Gd enhancement intensity measure of brain cysts in each stratum.

	Gadolinium enhancement (qualitative)			
	*Grade 0*	*Grade 1*	*Grade 2*	*Grade 3*
**Gadolinium enhancement intensity**				
**Untreated** *	18.30 (16.63–24.11)	34.43 (11.23–40.15)	35.31 (22.05–42.0)	0 (0–0)
**PZQ+ABZ 2d** **	0 (0–0)	30.96 (25.86–43.73)	34.51 (28.86–42.29)	41.37 (36.01–45.81)
**PZQ+ABZ 5d** ***	0 (0–0)	27.01 (17.95–41.85)	36.09 (16.49–48.49)	41.01 (24.95–51.54)

* r = 0.223, p = 0.0582

** r = 0.6098, p< 0.001

*** r = 0.3898, p<0.001

Spearman's correlation between quantitative and qualitative Gd enhancement.

### Correlation between EB staining and Gd enhancement in MRI

**Fig 4:**
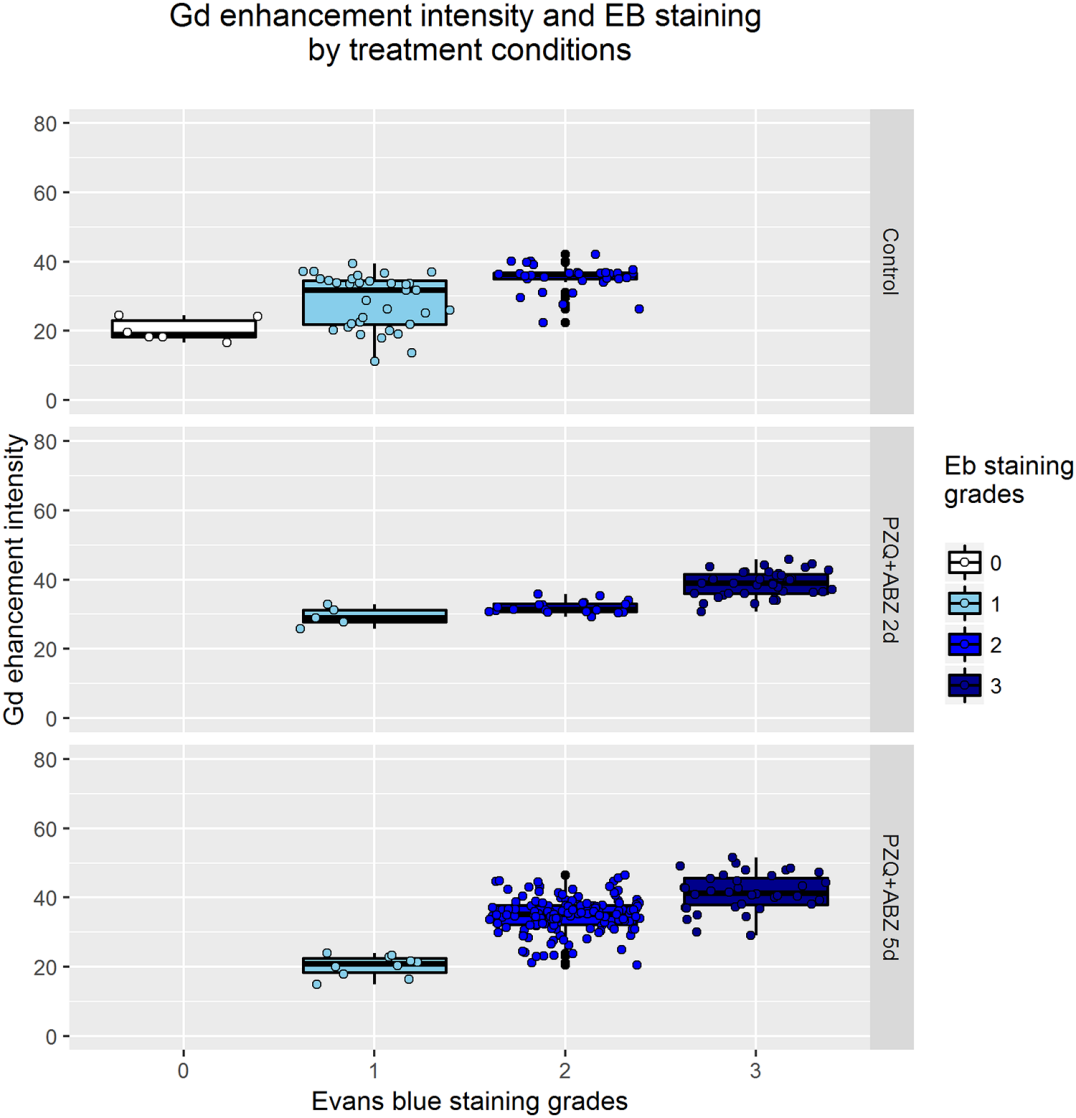
Quantitative assessment of perycistic Gd enhancement intensity on MRI by grades of EB staining and treatment group.

We analyzed the relationship between the different grades of EB staining and Gd enhancement intensity at the level of individual cysts per treatment group. Gd enhancement intensity had a significant tendency to increase with EB grades in all groups (Table 5, Fig 4).

**Table 5:** Correlation between EB staining and Gd enhancement intensity (quantitative) in MRI (n = 328 cysts). Data in each cell represent median and range for cysts in the stratum.

	Evans blue staining			
	*Grade 0*	*Grade 1*	*Grade 2*	*Grade 3*
**Gadolinium enhancement intensity**				
**Untreated** *	18.93 (16.63–24.56)	31.77 (11.23–39.51)	36.73 (22.44–42.0)	0 (0–0)
**PZQ+ABZ 2d** **	0 (0–0)	28.86 (25.86–32.84)	31.44 (29.31–35.75)	38.87 (30.75–45.81)
**PZQ+ABZ 5d** ***	0 (0–0)	20.88 (14.99–23.90)	35.07 (21.23–45.60)	41.25 (29.12–51.54)

* r = 0.5960, p<0.001

** r = 0.7860, p< 0.001

*** r = 0.5415, p<0.001

Spearman's correlation between quantitative Gd enhancement and EB staining.

### EB staining and Gd enhancement by cyst location

Since the inflammatory response can vary according to cyst location, we performed a stratified analysis between deep and superficial cysts (S3 Table). Considering the location of all cysts (328), there were 214 superficial and 114 deep cysts. Superficial cysts were more frequent in the PZQ+ABZ 5d group, (153 of 192), while deep and superficial cysts had similar frequency in the untreated (30 of 73) and the PZQ+ABZ 2d groups (31 of 63).

Within strata of treatment group, EB staining grades were significantly higher in deep cysts than in superficial cysts. These differences were statistically significant in the untreated group (p<0.001), where the majority of deep cysts were grade 2 (26/43, 60%) whereas superficial cysts were grade 1 (19/30, 64%). The same significant difference was observed in PZQ+ABZ 5d group (p<0.001), where the majority of deep cysts were grade 3 (30/39, 77%), whereas the superficial cysts were grade 2 (135/153, 88%). In PZQ+ABZ 2d group we found a marginal significance (p = 0.052) between deep (25/32, 78% were grade 3) and superficial cysts (17/31, 55% were grade 2). Gd enhancement intensity (quantitative measure) was significantly higher in deep cysts than in superficial cysts in the PZQ+ABZ 2d group (median 40.01 versus 33.02, p<0.001) and in the PZQ+ABZ 5d group (40.7 versus 35.07, p<0.001), but not in the untreated group (34.38 versus 33.57, p = 0.213).

### Histopathological assessment

Since the left brain hemispheres were reserved for molecular biology studies, only those cysts located in right brain hemispheres (n = 165) were available for microscopic assessment. Right hemisphere cysts were similar to those in the left hemisphere in terms of grade of EB staining or intensity of Gd enhancement (S4 Table). From these, 113 cysts (69%) from 11 pigs had available slides showing complete parasite structures (cyst wall and scolex with adjacent brain with immune response) and were thus used for histopathological studies in order to correlate inflammatory findings with the corresponding EB staining and MRI findings.

The distribution of inflammatory stages by cyst and treatment group was consistent with EB staining and Gd enhancement in the entire cyst population, with no extent of IS4 in untreated cysts and few extents of IS1 and IS2 in cysts from treated pigs (Table 6).

**Table 6:** Grading of inflammatory stage (histopathological assessment) by treatment group. Data in each cell represent median and range for cysts in the stratum.

		Untreated Median of IS (range)	PZQ+ABZ 2d Median of IS (range)	PZQ+ABZ 5d Median of IS (range)
**Inflammatory stages (n = 113)**	*IS 1*	0 (0–28)	0 (0–0)	0 (0–21)
	*IS 2*	60.5 (0–100)	0 (0–40)	0 (0–100)
	*IS 3*	37.5 (0–100)	90 (0–100)	78 (0–100)
	*IS 4*	0 (0–0)	10 (0–100)	5 (0–100)

**Fig 5:**
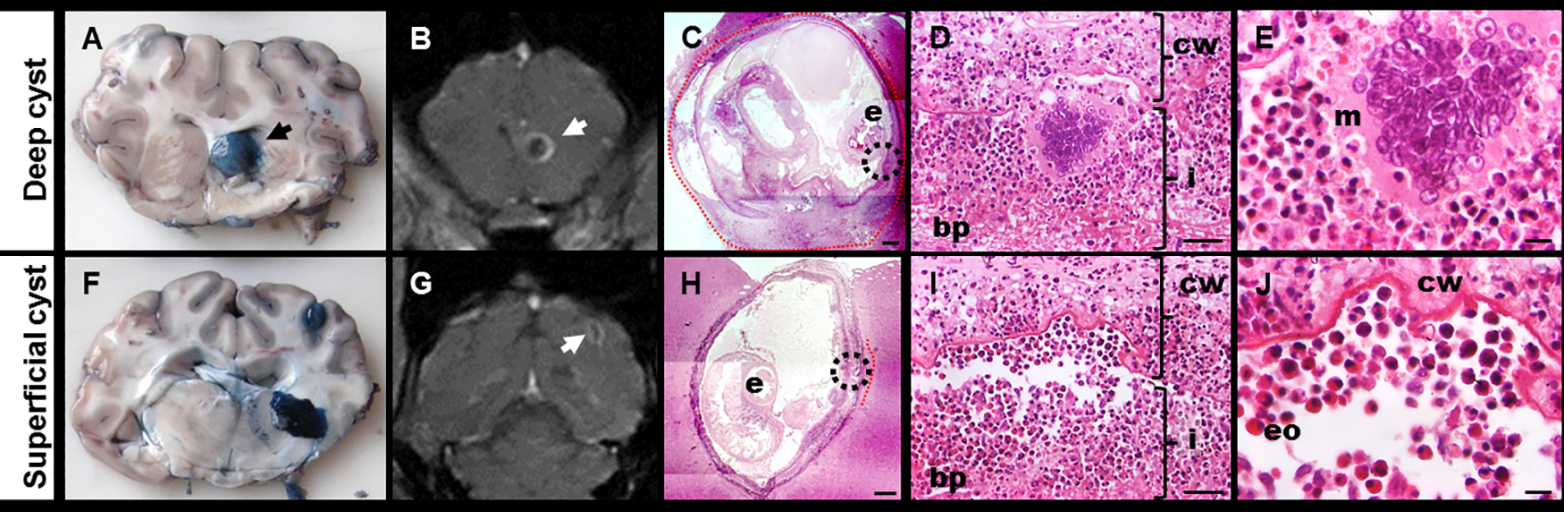
Correlation between EB staining, MRI findings and histology in deep and superficial cysts from PZQ+ABZ 5d group. **A** and **F** shows EB staining grade 3 cysts (black arrows) in macroscopic photographs. **B** and **G** show enhanced cysts (white arrows) in coronal T1-MRI. **C** and **H** show hematoxylin & eosin images (25X magnification; bar: 500 μm) where “e” indicates the scolex. **C** shows a deep cyst with 100% of IS4 extension (red line) whereas **H** shows a superficial cyst with a 10% of IS4 extension (red line). **D** and **I** are the 400X magnification (bar: 50 μm) of the circle (host-parasite interface) in **C** and **H,** respectively, and show IS4 (cw = cyst wall with damage, bp = brain parenchyma, i = infiltrate, mainly with eosinophils and lymphocytes). **E** and **J** show 1000x magnification (bar: 10 μm) of **D** and **I** (m = multinucleated giant cell, eo = eosinophils cells).

When assessed within this population of 113 cysts, we first correlated each inflammatory stage with EB staining and intensity of Gd enhancement in each treatment group. A positive and significant correlation was found only between intensity enhancement and IS4 (Spearman rank correlation; r = 0.363, p = 0.041) in the PZQ+ABZ 2d group. There were significant and positive correlations between IS3 and Gd enhancement intensity in the PZQ+ABZ 5d group (Spearman rank correlation; r = 0.358, p = 0.006) and between IS4 and EB staining (Spearman rank correlation; r = 0.578 p<0.001) and intensity enhancement (Spearman rank correlation; 0.50, p<0.001) in the PZQ+ABZ 5d. Also, IS4 was significantly higher in deep cysts than in superficial cysts in the PZQ+ABZ 5d group (median 10 versus 0, p<0.001) (Fig 5).

## Discussion

The study of inflammation due to degeneration of *T*. *solium* cysts (natural or induced by antiparasitic treatment) in NCC is limited by the lack of suitable animal models. Brain cysts can be found in the natural intermediate host, the pig, and studied by brain imaging (particularly MRI), or histopathology (macroscopically by assessing EB leakage, or by standard histological or immunohistochemistry techniques). MRI has been used in pigs naturally infected with NCC, providing important information on lesion characteristics (localization, parasite load, localization and stage of lesions, inflammation and perilesional edema, etc) [10–14], suggesting its usefulness to evaluate treatment schemes in the pig model. Disruption of BBB is associated with high levels of pro-inflammatory markers and inflammatory cell recruitment into pericystic tissue [6].

The injection of gadolinium as an MRI contrast agent reveals alterations in the integrity of the BBB in various neurological diseases (multiple sclerosis, strokes, acute ischemic brain injury, brain tumors, encephalomyelitis, etc.) [15–17]. The same mechanism is observed when the EB stain is used to evaluate alterations in BBB permeability. Our group has applied EB staining to mark BBB disruption in porcine NCC, demonstrating that treatment with praziquantel induces inflammation, cyst damage and BBB leakage at 2 and 5 days (48 and 120 h); we also have demonstrated that the BBB leakage is accompanied with expression of pro-inflammatory and immunoregulatory cytokines [5, 6]. In this study we evaluated the correlation between EB staining and contrast enhancement on MRI in regards to the inflammation and BBB leakage in the pig model of NCC. Our results show that EB extravasation around cysts as a marker of BBB disruption is equivalent to gadolinium enhancement on contrast T1 MRI, reflecting the location and intensity of pericystic inflammation areas. There was a strong correlation between both techniques and the histopathological findings; the location and intensity of gadolinium enhancement, as well as EB staining, reflected the degree and areas of histologically assessed inflammation. Using grades of EB staining and Gd enhancement also allowed for the understanding of the evolution of BBB disruption in the pericystic tissue as the immune response and local inflammation increased in response to anthelmintic treatment. Gd enhancement and EB staining increased markedly at 2 and 5 days post-treatment, which strongly correlated with greater perilesional inflammation. These results are consistent with other studies that evaluated the disruption of the BBB in brain tumors [18] and may explain the increase of symptoms in patients after the onset of antiparasitic treatment [19].

Chronic inflammation by *Taenia solium* cysts may cause increased angiogenesis in the granuloma around the cysts [20]. These neo-vessels may become more susceptible to vascular leakage after anthelmintic treatment due to the stimulus by pro-inflammatory molecules, thus the correlation of EB staining and Gd enhancement with the severe inflammatory response in NCC. In brain tumors there is a strong association between contrast enhancement and tumor neovascularization [21–23]. The association between brain inflammation and BBB disruption was also described in neurodegenerative diseases as Alzheimer’s disease and multiple sclerosis. In these diseases, the disruption of BBB integrity is associated with extravasation of proinflammatory molecules into the brain that cause damage in the nervous cells [24–25].

The inflammatory response varies according to the location of the parasite in the brain in relation to the parenchyma or the meninges; it is more evident in cysts surrounded by parenchyma [9]. In this study, deep (parenchymal) cysticerci showed stronger contrast enhancement in relation to areas where the inflammatory response was severe (IS3 or IS4), whereas superficial cysts frequently had a thin, weak enhancement signal and moderate or less severe inflammation in histological examinations (IS2 with some areas of IS3). This suggests enhancement is a marker of more intense and destructive inflammation as seen in other studies [26]. These findings are also consistent with our previous histological findings in the same model [9].

Our study had some limitations. First, the parasite load by pig was very variable, leading us to adjust analyses to account for this variability in the multivariate models. Second, the correlation with histopathology studies only used cysts located in the right hemisphere, although we found no reason to suspect any systematic differences between cysts in the left and right brain hemispheres [27], as seen in previous studies. Third, our analyses rely in two histological sections of cysts to determine the immune response; the two sections may not be representative of the inflammation around the whole cyst. Despite these limitations, the strong correlation between Gd enhancement on MRI and EB staining suggests that both methods are reliable in the evaluation of perilesional inflammation and BBB disruption in the porcine model of NCC and can be used alternatively or in combination.

## Supporting Information

S1 Table Grades of Evans blue staining in brain cysts per pigs by treatment group. Values represent numbers of brain cyst capsules in each pig.
(DOCX)

S2 Table Qualitative grade assessment of gadolinium enhancement in MRI of brain cysts per pigs by treatment group. Values represent numbers of brain cyst capsules in each pig.
(DOCX)

S3 Table Comparisons of Evans Blue staining and gadolinium enhancement intensity between cyst locations. Median values and ranges are shown for Gd enhancement intensity. EB staining grades are shown as number of cyst capsules and percentages. n = Number of cyst capsules. p* = Wilcoxon-Mann Whitney test used to compare EB staining grades and enhancement intensity between superficial and deep cysts in each treatment group.
(DOCX)

S4 Table Distribution of cyst characteristics between both hemispheres. Median values and ranges are shown for Gd enhancement intensity. EB staining grades are shown as number of cyst capsules and percentages. n = Number of cyst capsules. hem = hemisphere. p* = Wilcoxon-Mann Whitney test was used to compare EB staining grades and Gd enhancement intensity between cysts located in right and in left hemispheres in each treatment group.
(DOCX)
