# Supplementary material for: Perilesional Inflammation in Neurocysticercosis - Relationship Between Contrast-Enhanced Magnetic Resonance Imaging, Evans Blue Staining and Histopathology in the Pig Model
Source: PLoS Negl Trop Dis. 2016 Jul 26;10(7):e0004869. doi: 10.1371/journal.pntd.0004869 (PMC4961384; doi:10.1371/journal.pntd.0004869)
Supplement: S1 Table — Values represent numbers of brain cyst capsules in each pig. (DOCX) [file pntd.0004869.s001.docx]

**S1 Table. Grades of Evans blue staining in brain cysts per pigs by treatment group.** Values represent numbers of brain cyst capsules in each pig.

| **Treatment conditions** | | **Control**  **n (%)** | | | | **PZQ+ABZ 2d**  **n (%)** | | | | **PZQ+ABZ 5d**  **n (%)** | | | |
| --- | --- | --- | --- | --- | --- | --- | --- | --- | --- | --- | --- | --- | --- |
| **Pig ID** | | **1** | **2** | **3** | **4** | **5** | **6** | **7** | **8** | **9** | **10** | **11** | **12** |
| **Evans blue staining (Cyst capsules, %)** | ***Grade 0*** | 2  (4) | 4  (25) | 0 (0) | 0 (0) | 0 (0) | 0 (0) | 0 (0) | 0 (0) | 0 (0) | 0 (0) | 0 (0) | 0 (0) |
|  | ***Grade 1*** | 18  (40) | 12 (75) | 4 (36) | 0 (0) | 0 (0) | 0 (0) | 0 (0) | 5  (17) | 1  (1) | 0 (0) | 0 (0) | 9  (39) |
|  | ***Grade 2*** | 25  (56) | 0  (0) | 7 (64) | 1 (100) | 2 (20) | 3 (27) | 6 (46) | 8  (28) | 128 (84) | 0  (0) | 4 (31) | 12 (52) |
|  | ***Grade 3*** | 0 (0) | 0 (0) | 0 (0) | 0 (0) | 8 (80) | 8 (73) | 7 (54) | 16 (55) | 23  (15) | 4 (100) | 9 (69) | 2  (9) |
