# Supplementary material for: Perilesional Inflammation in Neurocysticercosis - Relationship Between Contrast-Enhanced Magnetic Resonance Imaging, Evans Blue Staining and Histopathology in the Pig Model
Source: PLoS Negl Trop Dis. 2016 Jul 26;10(7):e0004869. doi: 10.1371/journal.pntd.0004869 (PMC4961384; doi:10.1371/journal.pntd.0004869)
Supplement: S2 Table — Values represent numbers of brain cyst capsules in each pig. (DOCX) [file pntd.0004869.s002.docx]

**S2 Table. Qualitative grade assessment of gadolinium enhancement in MRI of brain cysts per pigs by treatment group.** Values represent numbers of brain cyst capsules in each pig.

| **Treatment conditions** | | **Control**  **n (%)** | | | | **PZQ+ABZ 2d**  **n (%)** | | | | **PZQ+ABZ 5d**  **n (%)** | | | |
| --- | --- | --- | --- | --- | --- | --- | --- | --- | --- | --- | --- | --- | --- |
| **Pig ID** | | **1** | **2** | **3** | **4** | **5** | **6** | **7** | **8** | **9** | **10** | **11** | **12** |
| **Gadolinium enhancement (Cyst capsules, %)** | ***Grade 0*** | 1  (2) | 3  (19) | 0 (0) | 0 (0) | 0 (0) | 0 (0) | 0 (0) | 0 (0) | 0 (0) | 0 (0) | 0 (0) | 0 (0) |
|  | ***Grade 1*** | 39 (87) | 10 (62) | 9 (82) | 0 (0) | 0 (0) | 5 (46) | 2 (15) | 3  (10) | 0 (0) | 0 (0) | 8 (61) | 14  (61) |
|  | ***Grade 2*** | 5  (11) | 3  (19) | 2 (18) | 1 (100) | 5 (50) | 4 (36) | 8 (62) | 22 (76) | 148 (97) | 1  (25) | 4 (31) | 8  (35) |
|  | ***Grade 3*** | 0 (0) | 0 (0) | 0 (0) | 0 (0) | 5 (50) | 2 (18) | 3 (23) | 4  (14) | 4  (3) | 3  (75) | 1  (8) | 1  (4) |
