# Supplementary material for: Perilesional Inflammation in Neurocysticercosis - Relationship Between Contrast-Enhanced Magnetic Resonance Imaging, Evans Blue Staining and Histopathology in the Pig Model
Source: PLoS Negl Trop Dis. 2016 Jul 26;10(7):e0004869. doi: 10.1371/journal.pntd.0004869 (PMC4961384; doi:10.1371/journal.pntd.0004869)
Supplement: S3 Table — Median values and ranges are shown for Gd enhancement intensity. EB staining grades are shown as number of cyst capsules and percentages. n = Number of cyst capsules. p* = Wilcoxon-Mann Whitney test used to compare EB staining grades and enhancement intensity between superficial and deep cysts in each treatment group. (DOCX) [file pntd.0004869.s003.docx]

**S3 Table. Comparisons of Evans Blue staining and gadolinium enhancement intensity between cyst locations.**

|  | **Control (n= 73)** | | **PZQ+ABZ 2d (n= 63)** | | **PZQ+ABZ 5d (n= 192)** | |
| --- | --- | --- | --- | --- | --- | --- |
|  | **Superficial**  **(n=30)** | **Deep**  **(n=43)** | **Superficial (n=31)** | **Deep**  **(n=32)** | **Superficial (n=153)** | **Deep**  **(n=39)** |
| **EB staining Grades** |  | | | | | |
| **0** | 4 (13%) | 2 (5%) | 0 (0%) | 0 (0%) | 0 (0%) | 0 (0%) |
| **1** | 19 (64%) | 15 (35%) | 0 (0%) | 5 (16%) | 10 (7%) | 0 (0%) |
| **2** | 7 (23%) | 26 (60%) | 17 (55%) | 2 (6%) | 135 (88%) | 9 (23%) |
| **3** | 0 (0%) | 0 (0%) | 14 (45%) | 25 (78%) | 8 (5%) | 30 (77%) |
| **p*** | <0.001 | | 0.052 | | <0.001 | |
| **Gd enhancement intensity** | 33.57  (11.23 -39.51) | 34.38  (18.34 - 42) | 33.02  (29.31 - 38.96) | 40.01  (25.86 - 45.81) | 35.07  (16.49 - 45.6) | 40.7  (23.05 - 51.54) |
| **p*** | 0.213 | | <0.001 | | <0.001 | |

Median values and ranges are shown for Gd enhancement intensity. EB staining grades are shown as number of cyst capsules and percentages.

n= Number of cyst capsules

p*= Wilcoxon-Mann Whitney test used to compare EB staining grades and enhancement intensity between superficial and deep cysts in each treatment group.
