# Supplementary material for: Perilesional Inflammation in Neurocysticercosis - Relationship Between Contrast-Enhanced Magnetic Resonance Imaging, Evans Blue Staining and Histopathology in the Pig Model
Source: PLoS Negl Trop Dis. 2016 Jul 26;10(7):e0004869. doi: 10.1371/journal.pntd.0004869 (PMC4961384; doi:10.1371/journal.pntd.0004869)
Supplement: S4 Table — Median values and ranges are shown for Gd enhancement intensity. EB staining grades are shown as number of cyst capsules and percentages. n = Number of cyst capsules. hem = hemisphere. p* = Wilcoxon-Mann Whitney test was used to compare EB staining grades and Gd enhancement intensity between cysts located in right and in left hemispheres in each treatment group. (DOCX) [file pntd.0004869.s004.docx]

**S4 Table. Distribution of cyst characteristics between both hemispheres.**

|  | **Control (n= 73)** | | **PZQ+ABZ 2d (n= 63)** | | **PZQ+ABZ 5d (n= 192)** | |
| --- | --- | --- | --- | --- | --- | --- |
|  | **Right hem (n= 30)** | **Left hem**  **(n= 43)** | **Right hem (n= 34)** | **Left hem**  **(n= 29)** | **Right hem (n= 101)** | **Left hem**  **(n= 91)** |
| **EB staining**  **Grades** |  | | | | | |
| **0** | 1 (3%) | 5 (12%) | 0 (0%) | 0 (0%) | 0 (0%) | 0 (0%) |
| **1** | 13 (44%) | 21 (49%) | 2 (6%) | 3 (10%) | 5 (5%) | 5 (5%) |
| **2** | 16 (53%) | 17 (39%) | 5 (15%) | 14 (48%) | 63 (62%) | 81 (89%) |
| **3** | 0 (0%) | 0 (0%) | 27 (79%) | 12 (42%) | 33 (33%) | 5 (6%) |
| **p*** | 0.166 | | 0.004 | | 0.001 | |
| **Gd enhancement intensity** | 36.04  (11.23 - 42) | 33.70  (13.70 - 39.71) | 38.17  (27.7 - 45.81) | 32.88  (25.86 – 42.29) | 35.07  (17.95 – 46.49) | 36.50  (14.99 - 51.54) |
| **p*** | 0.05 | | 0.001 | | 0.208 | |

Median values and ranges are shown for Gd enhancement intensity. And EB staining grades are shown as number of cyst capsules and percentages.

n= Number of cyst capsules

hem= hemisphere

p*= Wilcoxon-Mann Whitney test was used to compare EB staining grades and Gd enhancement intensity between cysts located in right and in left hemispheres in each treatment group.
